# The effect of guselkumab on inhibiting radiographic progression in patients with active psoriatic arthritis: study protocol for APEX, a Phase 3b, multicenter, randomized, double-blind, placebo-controlled trial

**DOI:** 10.1186/s13063-022-06945-y

**Published:** 2023-01-10

**Authors:** Christopher T. Ritchlin, Laura C. Coates, Philip J. Mease, Désirée van der Heijde, Jiao Song, Yusang Jiang, May Shawi, Alexa P. Kollmeier, Proton Rahman

**Affiliations:** 1 University of Rochester Medical Center, Rochester, USA; 2 Nuffield Department of Orthopaedics, Rheumatology and Musculoskeletal Sciences, University of Oxford, Oxford, UK; 3 Swedish Medical Center/Providence St. Joseph Health and University of Washington, Seattle, USA; 4 Leiden University Medical Center, Leiden, The Netherlands; 5 Janssen Research & Development, LLC, San Diego, USA; 6 Janssen Research & Development, LLC, Spring House, USA; 7 Immunology Global Medical Affairs, Janssen Pharmaceutical Companies of Johnson & Johnson, Horsham, USA; 8 Memorial University of Newfoundland, St. John’s, Canada

**Keywords:** Psoriatic arthritis, Spondyloarthritis, Randomized controlled trial, Radiographic data

## Abstract

**Background:**

Guselkumab, a fully human monoclonal antibody targeting the interleukin (IL)-23p19 subunit, is approved to treat adults with active psoriatic arthritis (PsA). In the Phase 3 DISCOVER-2 trial of 739 bilogico-naïve patients with active PsA, guselkumab 100 mg resulted in less radiographic progression, assessed via change from baseline in PsA-modified van der Heijde-Sharp (vdH-S) score, compared with placebo at week (W) 24 when given at W0, W4, and then every 4 weeks (Q4W) or Q8W. The least squares mean differences from placebo were -0.66 for guselkumab Q4W (*p*=0.011) and -0.43 for guselkumab Q8W (*p*=0.072). Reports suggest baseline C-reactive protein (CRP) and joint erosions are strongly prognostic of poor outcomes, especially radiographic progression, in PsA patients. We designed a trial (APEX) to further assess the effect of guselkumab on radiographic progression in patients with active PsA and risk factors for radiographic progression.

**Methods:**

Patients are eligible for APEX if they have had PsA for ≥6 months and active disease (≥3 swollen and ≥3 tender joints, CRP ≥0.3 mg/dL) despite prior therapy with conventional synthetic disease-modifying antirheumatic drugs, apremilast, and/or nonsteroidal anti-inflammatory drugs, with ≥2 joints with erosions on baseline radiographs (hands and feet). The primary and major secondary endpoints are the proportion of patients achieving ≥20% improvement in American College of Rheumatology response criteria (ACR20) response at W24 and change from baseline at W24 in PsA-modified vdH-S score, respectively. Sample sizes of 350/250/350 for guselkumab Q8W/guselkumab Q4W/placebo are expected to provide >99% power to detect significant differences in W24 ACR20 response rates for each guselkumab group vs placebo, as well as ≥90% (Q4W vs placebo) and ≥80% (Q8W vs placebo) power to detect a significant difference in PsA-modified vdH-S score change at W24. A Cochran-Mantel-Haenszel test and analysis of covariance will compare treatment efficacy for the primary and major secondary endpoints, respectively.

**Discussion:**

DISCOVER-2 findings informed the design of APEX, a Phase 3b study intended to further evaluate the impact of guselkumab in patients with active PsA and known risk factors for radiographic progression.

**Trial registration:**

This trial was registered at ClinicalTrials.gov, NCT04882098. Registered on 11 May 2021.

**Supplementary Information:**

The online version contains supplementary material available at 10.1186/s13063-022-06945-y.

## Background

Psoriatic arthritis (PsA) is a multi-faceted disease that impacts the joints, soft tissues, and skin [1]. The burden of disease can be severe, with some patients developing destructive arthritis leading to bony erosions and irreversible loss of joint architecture. PsA not only results in functional impairment and reduced quality of life, but is also associated with premature mortality [2–5]. Worsening of long-term functional impairment as well as progression of structural damage, which itself has been associated with a worsening of physical function [6], can be exacerbated by a delayed diagnosis of PsA; thus, a timely diagnosis (<6 months) and initiation of treatment are critical [7, 8]. Risk factors for structural damage progression that may be screened for and monitored include serum C-reactive protein (CRP); dactylitis count; enthesitis (yes/no),\; PsA subtype and duration; and numbers of joint erosions, joints with joint space narrowing (JSN), and swollen joints [9–13].

Structural damage of the joints, caused by the chronic inflammation underlying PsA, is associated with poorer outcomes for patients [14]. As a result, radiographic progression can be an important outcome measure in evaluating structural damage progression and assessing therapeutic agents, where inhibition of radiographic progression would improve treatment outcomes [14].

Guselkumab, a fully human monoclonal antibody targeting the interleukin (IL)-23p19 subunit, was first approved for adults with moderate-to-severe psoriasis and subsequently approved for treating the signs and symptoms of active PsA following two Phase 3 global studies, DISCOVER-1 (NCT03162796) [9] and DISCOVER-2 (NCT03158285) [10]. Both trials were randomized, placebo-controlled, double-blinded studies evaluating guselkumab 100 mg every 4 weeks (Q4W) and Q8W in adults with active PsA. DISCOVER-2 was the larger of the two studies (*N*=739) and enrolled only biologic-naïve patients with active PsA who had swollen and tender joint counts each ≥5 and serum CRP levels ≥0.6 mg/dL. In addition to the primary endpoint of the proportion of patients achieving ≥20% improvement in American College of Rheumatology criteria (ACR20) at week 24, DISCOVER-2 assessed change from baseline at week 24 in the PsA-modified van der Heijde-Sharp (vdH-S) score as a major secondary endpoint. The least squares (LS) mean difference in change from baseline in PsA-modified vdH-S score vs placebo was statistically significant in the guselkumab Q4W group (LS mean difference (−0.66, *p*=0.011); however, this difference did not reach statistical significance with the Q8W dosing regimen vs placebo (LS mean difference −0.43, *p*=0.072). Therefore, DISCOVER-2 may not have been sufficiently enriched for patients at risk for radiographic progression to adequately assess the effect of guselkumab Q8W on radiographic progression.

The Phase 3b APEX study described herein was designed to address these limitations of DISCOVER-2 and further assess the effects of guselkumab Q4W and Q8W on PsA outcomes, including clinical efficacy, radiographic progression, and health-related quality of life, in a biologic-naïve patient population enriched for those more likely to demonstrate radiographic progression.

## Methods/design

**Fig. 1:**
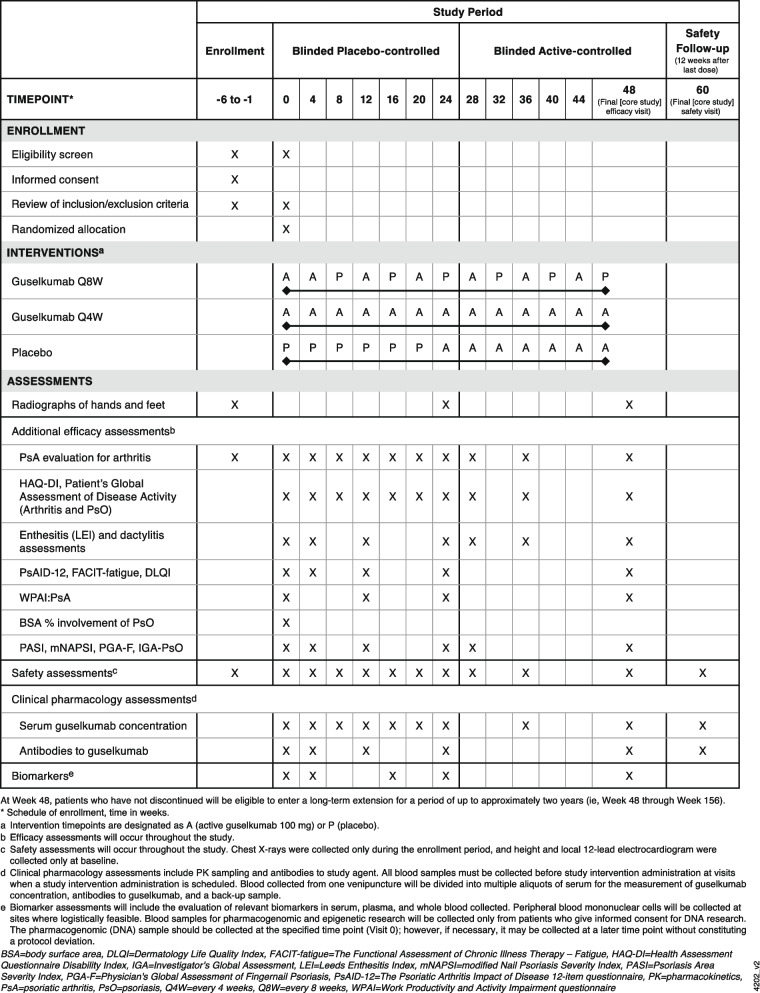
Standard protocol items: recommendation for interventional trials (SPIRIT) figure: trial visits and assessments during the core study

This APEX study protocol followed the SPIRIT reporting guidelines [15, 16] (Additional file 1). APEX is a 3 year (core study of 1 year followed by a long-term extension of 2 years) multicenter, randomized, double-blind, placebo-controlled, study of guselkumab in biologic-naïve patients with active PsA despite current standard therapies (i.e., conventional synthetic disease-modifying antirheumatic drugs [csDMARDs], nonsteroidal anti-inflammatory drugs [NSAIDs], and/or apremilast) (Fig. 1). Participant recruitment sites include private clinics and hospitals throughout Asia, Australia, Europe, and North America (https://clinicaltrials.gov/ct2/show/record/NCT04882098). Trial registration details can be found in Additional file 2.

### Assessments

Clinical efficacy assessments include the ACR response criteria, which define an ACR20 response as ≥20% improvement from baseline in both swollen (66 joints) and tender (68 joints) joint counts, and ≥20% improvement from baseline in three of the following assessments: patient pain (visual analog scale [VAS]), Patient’s Global Assessment of Disease Activity (arthritis, VAS), Physician’s Global Assessment of Disease Activity (VAS), Health-Assessment Questionnaire—Disability Index (HAQ-DI) [17], and serum CRP level [18]. Additional assessments include 28-joint disease activity score incorporating CRP (DAS28-CRP), Investigator’s Global Assessment of psoriasis (IGA) [19], and Psoriasis Area and Severity Index (PASI) [20]. Physical function will be evaluated using the HAQ-DI in a manner consistent with previous guselkumab randomized controlled trials [9, 10].

An independent joint assessor (IJA), other than the treating physician, will be designated at each study site to perform joint assessments (swollen and tender joint counts), as well as evaluations of enthesitis and dactylitis. The IJA will be blinded to patient data (except for joint assessments), will not discuss the patient’s clinical status with the patient or other site personnel during the joint assessment, and will have no other contact with the patient once randomized. Additionally, the IJA will not be permitted to review the patient’s medical records, the electronic case report form (eCRF), or any previous joint assessments.

Radiographs will be obtained to assess the progression of structural damage. To minimize unnecessary radiation exposure, it is recommended that the baseline radiographs of hands and feet be performed after the nonradiographic inclusion and exclusion criteria have been confirmed and the patient appears otherwise eligible to enter the study. Randomization will take place approximately 2–4 weeks following completion of baseline radiographs. Week 24 radiographs should be taken within ±2 weeks of the week 24 visit. If a patient discontinues prior to week 24, radiographs should be obtained at the week 24 visit. After week 24, radiographs will be collected at weeks 48, 96, and 156 (or at the time of study treatment discontinuation).

Radiographs will be evaluated by central independent readers and scored using the total vdH-S score modified for PsA by the addition of the distal interphalangeal joints of the hands and assessment of pencil in cup and gross osteolysis deformities [21, 22]. Erosion and JSN subscores (maximum: 320 and 208, respectively) will be summed to produce the total PsA-modified vdH-S score with a range of 0 to 528, with higher scores indicating more structural damage. The joint erosion subscore is a summary of erosion severity in 40 joints of the hands and 12 joints in the feet (score range: 0–320; 0 for no erosions to 5 in the hands and to 10 in the feet for extensive bone destruction). The JSN subscore summarizes the severity of JSN in 40 joints in the hands and 12 joints of the feet (score range: 0–208; 0 for no JSN to 4 for complete loss of joint space, bony ankylosis, or complete luxation).

Adverse events (AEs) will be reported by the patient (or, when appropriate, by a caregiver, surrogate, or the patient’s legally acceptable representative) for the duration of the study. Venous blood samples will be collected at regular intervals for the measurement of serum guselkumab concentrations and determining the presence of antibodies to guselkumab at the timepoints shown in Fig. 1. Blood samples for pharmacogenetic analyses will be obtained from patients who provide additional consent. Samples will be collected before study intervention at visits when a study intervention administration is scheduled.

### Objectives

To evaluate the efficacy of guselkumab treatment in patients with active PsA by assessing the reduction in signs and symptoms of PsA, the primary endpoint of APEX is the proportion of patients achieving an ACR20 response at week 24. To evaluate the inhibition of the progression of structural damage in patients with active PsA, the major secondary endpoint of APEX is change from baseline at week 24 in total PsA-modified van der Heijde-Sharp score. These and other secondary endpoints are summarized in Table 1.

**Table 1:** Study objectives and endpoints

Objectives	Endpoints
Primary	
• To evaluate the efficacy of guselkumab treatment in patients with active PsA by assessing the reduction in signs and symptoms of PsA.	Proportion of patients with ACR20 response at week 24: • ≥20% improvement from baseline in both swollen joint count (66 joints) and tender joint count (68 joints) AND • ≥20% improvement from baseline in three of the following assessments: ° Patient pain (VAS) ° PtGA (arthritis, VAS) ° PhGA (VAS) ° HAQ-DI ° Serum CRP level
Major secondary	
• To evaluate the inhibition of progression of structural damage in patients with active PsA.	Mean change from baseline in PsA-modified vdH-S score at week 24.
Other secondary	
• To evaluate the safety of guselkumab in patients with active PsA.	For the duration of the study, through week 60^a^: • Frequency and type of AEs, SAEs, reasonably related AEs, AEs leading to discontinuation of study intervention, infections, and injection-site reactions. • Frequency of laboratory abnormalities (chemistry, hematology), maximum toxicity CTCAE 5.0 grades.
• To evaluate the PK and immunogenicity of guselkumab in patients with active PsA.	For the duration of the study, through week 60^a^: • Mean/median serum guselkumab concentration. • Summary of incidence of antibodies to guselkumab.

Additional assessments - see Additional file 3

*ACR20* ≥20% improvement in American College of Rheumatology criteria, *AE* adverse event, *CRP* C-reactive protein, *CTCAE 5.0* Common Terminology Criteria for Adverse Events, *HAQ-DI* Health Assessment Questionnaire Disability Index, *Patient pain* patient’s assessment of pain, *PtGA* Patient’s Global Assessment of Disease Activity, *PhGA* Physician’s Global Assessment of Disease Activity, *PK* pharmacokinetics, *PsA* psoriatic arthritis, *SAE* serious adverse event, *VAS* visual analog scale, *vdH-S* van der Heijde-Sharp

^a^Through week 168 for patients who enter the long-term extension

Guselkumab safety will be evaluated through the frequency and type of AEs, serious AEs (SAEs), AEs leading to discontinuation of study intervention, infections, and injection-site reactions. Blood samples will be collected for serum chemistry and hematology assessments. The electronic Columbia suicide severity rating scale questionnaire [23, 24] will be used to capture any suicidal ideation or behavior. Malignancies and major adverse cardiovascular events will also be summarized. A complete list of study objectives and endpoints is provided in Additional file 3.

### Study population

The target study population for APEX is biologic-naïve adults with active PsA who demonstrated an inadequate response to standard therapies (e.g., csDMARDs, NSAIDs, and/or apremilast) and are at risk of radiographic progression. Several risk factors for radiographic progression were evaluated using historical radiographic data derived from placebo-treated PsA patients in prior Sponsor-conducted studies [9–13]. Among the factors evaluated (serum CRP level, dactylitis count, presence of enthesitis, PsA subtype, PsA duration, number of joints with erosion, number of joints with JSN, and number of swollen joints), baseline serum CRP level and number of joints with erosion had the strongest predictive value for identifying patients at risk of future radiographic progression (data on file, Janssen). Balancing study population enrichment with real-world PsA patient populations, CRP ≥0.3 mg/dL and ≥2 joint erosions on baseline radiographs of the hands and feet were chosen as key inclusion criteria for APEX (Table 2).

**Table 2:** Key inclusion and exclusion criteria

Inclusion criteria	Exclusion criteria
Age ≥18 years	Other inflammatory diseases, including: • rheumatoid arthritis • axial spondyloarthritis • systemic lupus erythematosus • Lyme disease
Active PsA despite previous csDMARD, NSAID, and/or apremilast, and/or NSAID therapy	
Diagnosis of PsA for ≥6 months prior to first administration of study intervention and meeting CASPAR criteria at screening	Previous biologic therapy for PsA or psoriasis
	Prior therapy with systemic immunosuppressants or apremilast within 4 weeks of study agent administration
Active PsA: • ≥3 swollen joints • ≥3 tender joints • CRP ≥0.3 mg/dL	Currently receiving ≥3 csDMARDs
≥2 joints with erosions on baseline radiographs of the hands and feet as determined by central read (2 readers and adjudicator if needed)	
≥1 of the following PsA subtypes: • distal interphalangeal joint involvement • polyarticular arthritis with absence of rheumatoid nodules • asymmetric peripheral arthritis • spondylitis with peripheral arthritis	
Active plaque psoriasis (≥1 plaque of ≥2 cm diameter and/or psoriatic nail changes)	

*CASPAR* Classification Criteria for Psoriatic Arthritis, *CRP* C-reactive protein, *csDMARD* conventional synthetic disease-modifying antirheumatic drug, *NSAID* nonsteroidal anti-inflammatory drug, *PsA* psoriatic arthritis

Methods of patient recruitment will include referral networks, site patient databases, posters in hospitals and waiting rooms, and advertising. Patient screening will be conducted by the study site investigators.

### Research ethics

This study will be conducted in accordance with principles of the Declaration of Helsinki, current International Conference on Harmonization and Good Clinical Practice (GCP) guidelines, applicable regulatory requirements, and Sponsor policy. The protocol and any modifications must be approved by the Institutional Review Board or Ethics Committee at each site and by local Health Authorities for each participating country. Prior to the conduct of any study-related procedures, investigators will collect written informed consent from all patients according to local requirements after the nature of the study has been fully explained; additional consent will be required for those patients who opt to participate in the voluntary pharmacogenomic testing.

Neither patients nor the public had a role in the design of APEX.

### Randomization and blinding

Central randomization will be implemented. Patients will be randomly assigned to 1 of 3 intervention groups based on a computer-generated randomization schedule prepared before the study by or under the supervision of the Sponsor. The randomization will be balanced using randomly permuted blocks and stratified by risk of radiographic progression, as assessed by baseline radiographic variability, corticosteroid use (yes/no), CRP level, and number of joints with erosions. The interactive web response system (IWRS) will assign a unique intervention code, which will dictate the intervention assignment and matching study intervention kit for the patient, who will be enrolled by the investigator at each study site.

Blinded intervention will be used to reduce potential bias during data collection and evaluation of endpoints. Guselkumab 100 mg and matching liquid placebo for guselkumab will be provided (Janssen Research & Development, LLC) in a single-use prefilled syringe assembled with the Ultrasafe PLUS^TM^ Passive Needle Guard. To maintain the study blind, study intervention containers will be labeled with non-identifying information only. The investigator will not be provided with patient randomization codes. Data that may potentially unblind the intervention assignment (i.e., serum concentrations, antibodies to guselkumab) will be handled with special care (e.g., special provisions, such as segregating the data in question from view by the investigators, clinical team, or others as appropriate until the time of database lock and unblinding) to maintain the integrity of the blind and to minimize the potential for bias.

At the week 24 database lock, the data will be unblinded to a limited number of Sponsor personnel for analysis of the primary and major secondary endpoints. Identification of Sponsor personnel who will have access to the unblinded patient-level data will be documented prior to unblinding. Investigative study sites and patients will remain blinded to initial treatment assignment until after the final database lock of the core study (week 48). The blind should not be broken until all patients have completed week 48 or discontinued prior to week 48 and the week 48 database lock has occurred. However, in the event of a medical emergency, the investigator will be able to identify the intervention by contacting the IWRS. The long-term extension (post-week 48) will be open-label.

#### Sample size justification

The planned study enrollment is approximately 950 patients, randomized 7:5:7 to subcutaneous guselkumab 100 mg at week 0, week 4, and then Q8W; subcutaneous guselkumab 100 mg Q4W; or placebo with prespecified crossover to guselkumab 100 mg Q4W at week 24. Sample sizes were based on week 24 ACR20 response rates (64.6%, 63.7%, and 33.1% for guselkumab Q8W, guselkumab Q4W, and placebo, respectively) and mean (SD) changes from baseline at week 24 in total PsA-modified vdH-S scores (0.45 [2.38], 0.25 [2.52], and 0.90 [3.14] for guselkumab Q8W, guselkumab Q4W, and placebo, respectively) (data on file) from DISCOVER-2 [10]. Assumptions based on these data were adjusted for differences in enrichment criteria between DISCOVER-2 and APEX. Assuming ACR20 response rates of 60% in the guselkumab groups and 35% in the placebo group for APEX, sample sizes of 350/250/350 for guselkumab Q8W/guselkumab Q4W/placebo are expected to provide >99% power to detect significant differences in ACR20 response rates at week 24 for each guselkumab group vs. placebo at a 2-sided significance level of α=0.05 using a 2-sided chi-square test. Assuming changes from baseline at week 24 in PsA-modified vdH-S scores of 0.45 (3.1), 0.25 (3.1), and 1.13 (3.2) for guselkumab Q8W, guselkumab Q4W, and placebo, respectively, these sample sizes are also expected to provide ≥90% (Q4W vs placebo) and ≥80% (Q8W vs placebo) power to detect a significant difference in change from baseline in PsA-modified vdH-S score at week 24 at a 2-sided significance level of α=0.05.

### Study design

Patients will be randomized to 1 of the 3 treatment groups as described above. At week 16, all patients who qualify for early escape (<20% improvement from baseline in both tender and swollen joint counts) will continue the study agent and dosing regimen to which they were randomized but will be allowed to initiate or increase the dose of one of the permitted concomitant medications, up to the maximum approved dose, at the investigator’s discretion. Concomitant use of stable doses of NSAIDs, oral corticosteroids (equivalent to ≤10 mg of prednisone), and up to 2 csDMARDs, limited to methotrexate (≤25 mg/week), sulfasalazine (≤3g/day), hydroxychloroquine (≤400 mg/day), or leflunomide (≤20 mg/day), will be permitted.

**Fig. 2:**
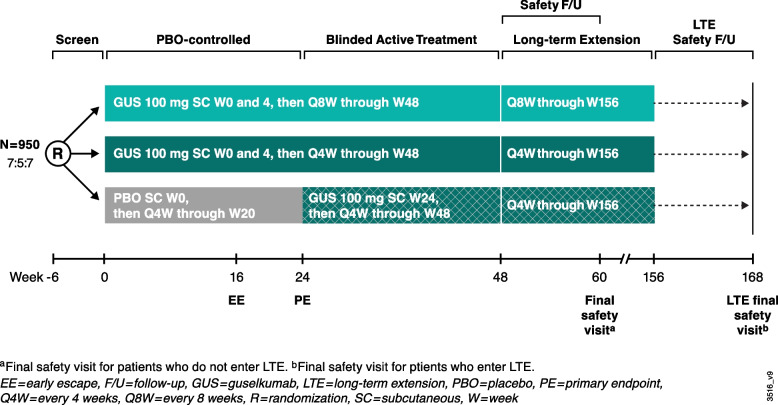
APEX study schema. The APEX trial includes a 6-week screening period, 24-week placebo-controlled period, 24-week active treatment period, open-label long-term extension period from W48 through W156, and safety follow-up through W60 (final safety visit for patients who do not enter LTE) or W168 (final safety visit for patients who enter LTE). EE if <20% improvement from baseline in tender and swollen joint counts at W16, patients may initiate or increase dose of one permitted concomitant medication up to the maximum allowed dose

There will be 5 phases in this 3-year study: a screening phase of up to 6 weeks, a double-blind placebo-controlled phase (week 0 to week 24), a double-blind active treatment phase (week 24 to week 48), an open-label long-term extension (week 48 through week 156), and a safety follow-up (12 weeks from the last administration of study intervention (Fig. 2).

### Intervention

Both guselkumab dosing regimens, Q4W and Q8W, have demonstrated clinically meaningful efficacy and with an acceptable safety profile in patients with active PsA [9, 10, 25]. APEX is expected to provide additional clinical safety and efficacy data in patients with PsA, especially toward evaluating the inhibition of progression of structural damage. Inclusion of the guselkumab 100 mg Q4W and Q8W dosing regimens will allow a relative benefit-risk assessment in this regard.

Although guselkumab has been approved for patients with active PsA in several countries, the use of a placebo control is necessary to establish the frequency and magnitude of changes in clinical efficacy and radiographic endpoints that may occur in the absence of active intervention, particularly in this PsA population enriched for patients more likely to experience radiographic progression. The placebo selected for this study is identical in appearance to guselkumab.

All patients will receive study injections at 4-week intervals: patients randomized to the guselkumab Q4W group will receive only guselkumab 100 mg; patients randomized to the guselkumab Q8W group will receive guselkumab 100 mg at weeks 0 and 4 then Q8W, with placebo injections (Q8W beginning at week 8) to maintain the blind; and patients randomized to placebo will receive only placebo injections at weeks 0, 4, 8, 12, 16, and 20, followed by guselkumab 100 mg Q4W beginning at week 24 (Fig. 1). Patients who discontinue study intervention should be encouraged to return for all remaining core study visits; it is particularly important for patients to return for all visits through week 24. Radiographs of the hands and feet should still be performed at week 24 for patients who discontinue study agent prior to week 24.

### Statistical methods

In general, descriptive statistics, such as mean, standard deviation (SD), median, interquartile range, minimum, and maximum for continuous variables, and counts and percentages for discrete variables, will be used to summarize most data. With the primary endpoint of ACR20 response at week 24 and the major secondary endpoint of change from baseline at week 24 in PsA-modified vdH-S score, and two treatment comparisons (guselkumab Q4W versus placebo and guselkumab Q8W versus placebo) per endpoint, APEX will test four hypotheses. For the primary analysis of the primary endpoint, treatment comparisons will be performed using a Cochran-Mantel-Haenszel (CMH) test. For sensitivity analyses and other secondary binary endpoints, a Generalized Linear Mixed Model will be used. Treatment group comparisons for the major secondary endpoint will be performed using analysis of covariance (ANCOVA), and a mixed model for repeated measures will be used for other secondary continuous endpoints. In general, statistical testing will be performed using 2-sided tests. The overall type I error of the four hypotheses will be controlled at a significance level of ≤0.05 and will be tested in a fixed sequence. For the primary endpoint, if guselkumab Q4W versus placebo is significant at a 2-sided α-level of 0.05, the study will be considered positive, and the second test of guselkumab Q8W vs placebo will be performed. The two hypotheses for the major secondary endpoint of change from baseline in PsA-modified vdH-S score at week 24 will be tested in a fixed sequence if both hypotheses of the primary endpoint are statistically significant.

#### Estimands

The primary endpoint, ACR20 response at week 24, will be analyzed based on the adjusted composite estimand, defined by the following components: (1) population (biologic-naïve adults with active PsA); (2) treatment (placebo or guselkumab); (3) variable (week 24 ACR20 composite binary response, where response is defined as achievement of ACR20 response at week 24 where intercurrent events [ICEs] 1–5 (Table 3) did not occur before week 24); (4) ICEs (Table 3); and (5) population level summary.

**Table 3:** ICEs for the analyses of the primary and major secondary endpoints

1. Discontinued study intervention injections due to any reason except due to Natural Disaster or Major Disruption^a^	
2. Initiated or increased the dose of csDMARDs or oral corticosteroids over baseline for PsA^a^	
3. Initiated protocol prohibited medications/therapies for PsA^a^	
4. Discontinued study intervention injections due to Natural Disaster or Major Disruption^b^	
5. Severe treatment non-compliance due to study site access restrictions, defined as ≥2 doses of study intervention missed due to Natural Disaster or Major Disruption^b^	

*csDMARD* conventional synthetic disease-modifying antirheumatic drug, *ICE* intercurrent event, *Major Disruption* disruption in Ukraine and neighboring countries/territories beginning February 24, 2022, *Natural Disaster* site closure, site access restrictions, or lockdowns caused by COVID-19, *PsA* psoriatic arthritis

^a^Patients meeting criteria for ICEs 1–3 are considered nonresponders for the primary endpoint (composite strategy). These ICEs are considered irrelevant to the major secondary endpoint (treatment policy strategy)

^b^ICEs 4 and 5 follow the hypothetical strategy (see the “ICE strategy and missing data” section)

The major secondary endpoint’s main analysis is based on the adjusted treatment policy estimand, which has components similar to those of the adjusted composite estimand, with the exception of the variable and the population level summary (difference in mean changes between guselkumab Q4W and placebo and between guselkumab Q8W and placebo).

#### ICE strategy and missing data

For the primary endpoint analyzed under the adjusted composite estimand, occurrence of ICEs prior to week 24 that follow the composite strategy will be considered as treatment failure, and such patients will be treated as nonresponders at week 24 regardless of the observed ACR20 response status, while for ICEs that follow the hypothetical strategy (unplanned changes to study conduct as a result of Natural Disaster [site closure, site access restrictions, or lockdowns caused by COVID-19] or Major Disruption [disruption in Ukraine and neighboring countries/territories beginning February 24, 2022]), data observed after meeting ICE criteria will not be used and will be considered missing at random (MAR) and imputed using multiple imputations on individual ACR components. Patients with missing data not related to Natural Disaster or Major Disruption will be conservatively imputed as ACR20 nonresponders using nonresponder imputation (NRI). Efficacy analyses will be performed using the modified full analysis set (i.e., all randomized patients, excluding those from sites unable to support key study operations due to Natural Disaster or Major Disruption. 

The treatment difference between each guselkumab group and placebo will be tested using a CMH test (stratified by the randomization strata levels from each MI dataset) with Wilson-Hilferty transformation [26] applied. The magnitude of the effect will be estimated by the difference in ACR20 response rates between the guselkumab and placebo groups with the 95% confidence interval (CI) calculated based on Wald statistics. To evaluate the robustness of the primary endpoint analysis results, sensitivity analyses include but are not limited to the exhaustive scenario tipping point analysis, which will be performed by varying the amount of NRI for missing data for both guselkumab and placebo. Subgroup analyses will be performed to evaluate consistency in the primary efficacy endpoint by demographic characteristics, baseline disease characteristics, and baseline medication use. Interaction tests between the subgroups and treatment group will also be conducted when a subgroup has at least two categories. If the number of patients in a subgroup is <10, subgroups may be pooled for analyses.

The adjusted treatment policy estimand (major secondary endpoint) is defined by five components similarly to the primary endpoint (described above), with differences in variable (change from baseline in PsA-modified vdH-S score at week 24 where ICEs 4 and 5 (Table 3) do not occur) and population level summary (difference in mean changes between guselkumab Q4W and placebo and between guselkumab Q8W and placebo). Note that for this estimand, ICEs 1–3 will follow the treatment policy strategy, which is to use all observed data collected for the endpoint; ICEs 4–5 will use the hypothetical strategy similar to the adjusted composite estimand. Change from baseline in PsA-modified vdH-S score at week 24 will be analyzed based on central readings from the first reading session (radiographs obtained at week 0 and week 24). Missing data will be imputed using MI under the assumption that data are MAR. Treatment comparisons for each imputation dataset will be based on an ANCOVA model adjusted for baseline score and possible other covariates to be specified in the Statistical Analysis Plan. The LS mean difference in change from baseline in PsA-modified vdH-S score and corresponding 95% CI will be provided.

#### Safety, immunogenicity, and pharmacokinetics analyses

All patients who receive ≥1 study intervention (complete or partial) will be included in the safety analyses. Analyses of AEs will include the incidence of AEs, SAEs, infections, and injection site reactions. All patients who receive ≥1 study intervention (complete or partial) and who have ≥1 sample obtained after the first study intervention administration will be included in the immunogenicity analyses. All patients who receive ≥1 study intervention (complete) and who have ≥1 valid blood sample drawn after the first study intervention administration will be included in the pharmacokinetics analyses.

### Oversight and monitoring

A Trial Steering Committee of independent members will supervise this study. Steering committee objectives are to (1) provide practical advice on strategy and direction of trial; (2) provide clinical expertise and advice on best clinical study parameters (program design, population, endpoints, etc.); (3) participate in data review, analysis, and interpretation of trial results; and (4) guide/suggest additional analyses to inform clinical practice. Steering Committee meetings occur via teleconference on an as-needed basis and will be attended by both Steering Committee members and employees or representatives of the study Sponsor. Meetings are facilitated through the Sponsor in consultation with the Steering Committee.

As part of study site monitoring, Sponsor personnel/designees will monitor study site conduct to ensure adherence to the protocol and GCP using a combination of monitoring techniques including central, remote, and on-site monitoring. On-site monitoring visits will occur as frequently as necessary, the first of which will be as soon as possible after patient enrollment. At these visits, eCRF and source document (hospital/clinic/physician’s office medical records) data will be compared for consistency. The nature and location of all source documents will be documented and made accessible to the Sponsor study site contact for verification purposes. During all monitoring visits, relevant study site personnel will be available, source documents will be accessible, and a suitable environment will be provided for review of study-related documents. The monitor will meet with the study investigator on a regular basis during the study to provide feedback on study conduct. The study Sponsor, including the study responsible physician, scientist, and operations team, is responsible for daily oversight of APEX.

As guselkumab has now been approved by several regulatory agencies (including when given Q4W in the EU) for use in patients with PsA, a Data and Safety Monitoring Board/Independent Data Monitoring Committee is not planned for the post-marketing APEX study. APEX maintains standard blinded medical monitoring procedures.

### Frequency and procedures for auditing trial conduct

To ensure accuracy and reliability of data, qualified investigators and appropriate study sites have been selected for this study. Protocol procedures and eCRF guidelines are reviewed with investigators and study site personnel, and clinical laboratory data will be transmitted directly to the Sponsor’s database and verified for accuracy and consistency. Representatives of the Sponsor's clinical quality assurance department may visit the study site at any time during or after completion of the study to conduct an audit of the study in compliance with regulatory guidelines and company policy to review study records. The Sponsor will also review the eCRF for accuracy and completeness, and discrepancies will be resolved with the appropriate investigator.

Patient privacy guidelines and applicable laws will be followed. Similar auditing procedures may also be conducted by agents of any regulatory body, either as part of a national GCP compliance program or to review the results of this study in support of a regulatory submission.

## Discussion

Structural damage can carry a significant impact for PsA patients, with associated impairments in physical function and quality of life; damage is irreversible and can be rapid in some patients [14]. Typically measured by radiographs, existing structural damage is a prognostic factor that can influence treatment choices [27] and is an important consideration when evaluating the role of a therapeutic agent in improving the signs and symptoms of PsA [28, 29].

Not all PsA patients will respond to the same treatment approach. The European Alliance of Associations for Rheumatology and the Group for Research and Assessment of Psoriasis and Psoriatic Arthritis recommend an individualized treatment strategy influenced by various factors across PsA disease domains (peripheral arthritis, axial arthritis, enthesitis, dactylitis, skin, and nail disease), previous therapies, and prognostic factors such as structural damage and comorbidities. Such treatments may include csDMARDs and biologics (targeting TNF, IL-17A, IL-23, or IL-12/23) [27, 30]. Although an inhibition of radiographic progression in patients with PsA has been associated with the use of TNF inhibitors [31, 32], reports have also shown a high rate of discontinuation among patients receiving them [33]. There remains, then, a continued need for effective therapies that inhibit radiographic damage in PsA patients [27].

The DISCOVER-2 trial provided data indicating the efficacy of guselkumab, a fully human IL-23 inhibitor, in biologic-naïve PsA patients, and assessed the impact of treatment on radiographic progression [10]. At week 24 in DISCOVER-2, patients receiving guselkumab Q4W exhibited significantly less structural damage progression than those receiving placebo; numerically less damage with guselkumab Q8W than placebo was also observed [10]. Low rates of radiographic progression were further observed through 1 and 2 years among guselkumab-treated patients receiving either the Q4W or Q8W dosing regimen [34, 35]. Patients achieving clinical response across several joint-specific and global measures of disease activity or who achieved normalized physical function at week 100 had lower mean changes in total PsA-modified vdH-S scores compared with nonresponders [35]. Taken together, improvements in signs and symptoms of PsA via IL-23p19 inhibition with guselkumab are associated with low levels of radiographic progression through 2 years in biologic-naïve PsA patients. Guselkumab is the first IL-23 inhibitor to demonstrate such long-term findings in such a PsA patient population.

Findings from DISCOVER-2 informed the design of APEX, a Phase 3b study intended to further evaluate the impact of selectively targeting the IL-23p19 subunit with guselkumab in patients with active PsA and known risk factors for radiographic progression. APEX is intended to address limitations specific to DISCOVER-2, notably the potentially underpowered nature of the study to demonstrate inhibition of structural damage with both guselkumab dosing regimens. The study duration of 3 years will also allow for a more thorough evaluation of structural damage progression, which accumulates over time and, as a result, requires long-term assessment [14]. In selecting a biologic-naïve population with ≥2 joints with erosions at baseline, APEX will focus on patients more likely to experience radiographic progression. Further, a larger sample size will increase the power to detect a treatment effect, and independent, central readings will reduce variability surrounding the change from baseline in PsA-modified vdH-S score. With this enriched population and longer study duration, APEX is intended to demonstrate the efficacy and continued low rates of radiographic progression with guselkumab in patients with active PsA.

## Trial status

First patient was dosed on 1 JUL 2021. APEX has an expected study completion date of 21 JUL 2027. Protocol amendment 2, 4 MAY 2022.

## Supplementary Information

**Additional file 1.** APEX SPIRIT checklist.

**Additional file 2.** APEX trial registration data.

**Additional file 3.** Objectives and endpoints of APEX.

## Data Availability

The data sharing policy of Janssen Pharmaceutical Companies of Johnson & Johnson is available at https://www.janssen.com/clinical-trials/transparency. As noted on this site, requests for access to the study data can be submitted through Yale Open Data Access [YODA] Project site at http://yoda.yale.edu.

## Abbreviations

ACR: American College of Rheumatology
AE: Adverse event
ANCOVA: Analysis of covariance
CASPAR: ClASsification criteria for Psoriatic ARthritis
CI: Confidence interval
CMH: Cochran-Mantel-Haenszel
CRP: C-reactive protein
csDMARD: Conventional synthetic disease-modifying anti-rheumatic drug
CTCAE: Common Terminology Criteria for Adverse Events
DAS-28: 28-joint disease activity score
DMARD: Disease-modifying antirheumatic drug
EE: Early escape
GCP: Good Clinical Practice
GUS: Guselkumab
HAQ-DI: Health Assessment Questionnaire-Disability Index
ICE: Intercurrent event
IGA: Investigator’s Global Assessment
IJA: Independent joint assessor
IL: Interleukin
IWRS: Interactive web response system
JSN: Joint space narrowing
LS: Least squares
LTE: long-term extension
MAR: Missing at random
MI: Multiple imputation
NRI: Nonresponder imputation
NSAID: Nonsteroidal anti-inflammatory drug
PASI: Psoriatic Area and Severity Index
PBO: Placebo
PhGA: Physician’s Global Assessment
PK: Pharmacokinetic
PsA: Psoriatic arthritis
PtGA: Patient’s Global Assessment
Q4W: Every 4 weeks
Q8W: Every 8 weeks
SAE: Serious adverse event
SD: Standard deviation
SPIRIT: Standard Protocol Items: Recommendation for Interventional Trials
TNFi: Tumor necrosis factor inhibitor
vdH-S: van der Heijde-Sharp (score)
W: Week
