# Supplementary material for: The effect of guselkumab on inhibiting radiographic progression in patients with active psoriatic arthritis: study protocol for APEX, a Phase 3b, multicenter, randomized, double-blind, placebo-controlled trial
Source: Trials. 2023 Jan 10;24:22. doi: 10.1186/s13063-022-06945-y (PMC9830619; doi:10.1186/s13063-022-06945-y)
Supplement: Supplementary file 2 — Additional file 2. APEX trial registration data. [file 13063_2022_6945_MOESM2_ESM.docx]

| **Additional File 2. APEX trial registration data.** | |
| --- | --- |
| Data category | Information |
| Primary registry and trial identifying number | ClinicalTrials.gov  NCT04882098 |
| Date of registration in primary registry | 11, May 2021 |
| Secondary identifying numbers | CR108957, 2020-004981-20, CNTO1959PSA3004 |
| Source of monetary or material support | Janssen Scientific Affairs, LLC |
| Contact for public queries | Phone: 844-434-4210  Email: [Participate-In-This-Study@its.jnj.com](mailto:Participate-In-This-Study@its.jnj.com) |
| Contact for scientific queries | Phone: 844-434-4210  Email: [Participate-In-This-Study@its.jnj.com](mailto:Participate-In-This-Study@its.jnj.com) |
| Public title | A Study of Guselkumab in Participants With Active Psoriatic Arthritis (APEX) |
| Scientific title | A Phase 3b, Multicenter, Randomized, Double-blind, Placebo-controlled Study Evaluating the Efficacy and Safety of Subcutaneously Administered Guselkumab in Improving the Signs and Symptoms and Inhibiting Radiographic Progression in Participants With Active Psoriatic Arthritis |
| Countries of recruitment | United States, Australia, Belarus (suspended), Bosnia and Herzegovina, Bulgaria, Canada, Croatia, Czechia, Estonia, Georgia, Germany, Hungary, Israel, Italy, Latvia, Lithuania, Philippines, Poland, Russia, Serbia, Slovenia, Spain, Taiwan, Ukraine |
| Health condition(s) or problem(s) studied | Psoriatic arthritis |
| Intervention(s) | Active comparator: 100 mg subcutaneous guselkumab Q4W or Q8W  Placebo comparator: matching liquid placebo for guselkumab Q4W through W20 |
| Key inclusion and exclusion criteria | Ages eligible for study: 18 years and older  Sexes eligible for study: All  Accepts healthy volunteers: No  Inclusion criteria: See Table 2  Exclusion criteria: See Table 2 |
| Study type | Interventional  Allocation: Randomized, parallel assignment intervention model, double-blind masking (participant, investigator)  Primary purpose: Treatment  Phase 3b |
| Date of first enrollment | 1, July 2021 |
| Target sample size | 950 participants |
| Recruitment status | Recruiting |
| Primary outcome(s) | See Table 1 |
| Key secondary outcomes | See Table 1 |
