# Supplementary material for: The effect of guselkumab on inhibiting radiographic progression in patients with active psoriatic arthritis: study protocol for APEX, a Phase 3b, multicenter, randomized, double-blind, placebo-controlled trial
Source: Trials. 2023 Jan 10;24:22. doi: 10.1186/s13063-022-06945-y (PMC9830619; doi:10.1186/s13063-022-06945-y)
Supplement: Supplementary file 3 — Additional file 3. Objectives and endpoints of APEX. [file 13063_2022_6945_MOESM3_ESM.docx]

| **Additional File 3. Objectives and Endpoints of APEX** | |
| --- | --- |
| Objective | Endpoint |
| Primary |  |
| - To evaluate the efficacy of guselkumab treatment in patients with active psoriatic arthritis (PsA) by assessing the reduction in signs and symptoms of PsA. | - Proportion of patients with American College of Rheumatology (ACR) 20 response at Week 24 |
| Major Secondary |  |
| - To evaluate the inhibition of progression of structural damage in patients with active PsA. | - Change from baseline in PsA modified van der Heijde-Sharp (vdH-S) score at Week 24. |
| Other Secondary |  |
| - To evaluate the safety in patients with active PsA. | - Frequency and type of adverse events (AEs), serious adverse events (SAEs), reasonably related AEs, AEs leading to discontinuation of study intervention, infections, infusion reactions, and injection-site reactions. - Laboratory abnormalities (chemistry, hematology), maximum toxicity (Common Terminology Criteria for Adverse Events [CTCAE 5.0]) grades. |
| - To evaluate the PK and immunogenicity in patients with active PsA | - Serum guselkumab concentration - Incidence of antibodies to guselkumab |
| Other | |
| Endpoints Related to Reduction of Signs and Symptoms and Physical Function   - Proportions of patients who achieve ACR20, ACR50, and ACR70 responses by visit over time   through Week 156.   - Proportion of patients who maintain an ACR20/50/70 response at Week 48 and Week 156 among the patients who achieved an ACR20/50/70 response at Week 24. - Change, and percent change from baseline in ACR components by visit over time through Week 156. - Change from baseline in HAQ-DI score by visit over time through Week 156. - Proportion of patients who achieve a clinically meaningful improvement (a ≥0.35 improvement from baseline) in HAQ-DI score by visit over time through Week 156 among those patients with HAQ-DI score ≥0.35 at baseline. - Proportion of patients who maintain a HAQ-DI response (i.e., ≥0.35 improvement from baseline in HAQ-DI score) at Week 48 and Week 156 among the patients who achieved a HAQ-DI response at Week 24. - Proportion of patients who achieve a DAS28 (CRP) response/remission by visit over time through Week 156. - Change from baseline in DAS28 (CRP) by visit over time through Week 156. - Proportion of patients who achieve a response based on mPsARC by visit over time through Week 156. - Proportion of patients with resolution of enthesitis (LEI)/dactylitis by visit over time through Week 156 among the patients with enthesitis/dactylitis at baseline. - Change from baseline in enthesitis (based on LEI)/dactylitis score by visit over time through Week 156 among the patients with enthesitis/dactylitis at baseline. - Change from baseline in WPAI, mCPDAI, and DAPSA scores by visit over time through Week 156. - Proportion of patients who achieve DAPSA low disease activity, DAPSA remission, MDA, or VLDA by visit over time through Week 156.   **Endpoints Related to Skin Disease**   - Proportions of patients who achieve a PASI 75/90/100 score from baseline by visit over time through Week 156 among the patients with ≥3% BSA psoriatic involvement and an IGA score of ≥2 (mild) at baseline. - Proportion of patients with a psoriasis response of an IGA score of 0/1 (i.e., an IGA-psoriasis score of 0 [cleared]/1 [minimal] AND ≥2-grade reduction from baseline), or IGA score of 0 by visit over time through Week 156 among the patients with ≥3% BSA psoriatic involvement and an IGA score of ≥2 (mild) at baseline. - Change and percent change from baseline in PASI score by visit over time through Week 156 among the patients with ≥3% BSA psoriatic involvement and an IGA score of ≥2 (mild) at baseline. - Proportion of patients who achieve both PASI 75 and ACR20 responses, or both PASI 75 and mPsARC by visit over time through Week 156 among the patients with ≥3% BSA psoriatic involvement and an IGA score of ≥2 (mild) at baseline. - Proportion of patients who achieve a DLQI score of 0/1 by visit through Week 156 among patients with baseline DLQI score >1 and with ≥3% BSA psoriatic involvement and an IGA score of ≥2 (mild) at baseline - Proportion of patients who achieve ≥5-point improvement from baseline in DLQI score by visit through Week 156 among the patients with baseline DLQI score ≥5 and with ≥3% BSA psoriatic involvement and an IGA score of ≥2 (mild) at baseline. - Change from baseline in DLQI score by visit through Week 156 among the patients with ≥3% BSA psoriatic involvement and an IGA score of ≥2 (mild) at baseline.   **Endpoints Related to Psoriasis of the Nails**   - Percent change from baseline in total fingernail mNAPSI score by visit over time through Week 156 among the patients with total fingernail mNAPSI score >0 at baseline. - Proportions of patients who achieve total fingernail mNAPSI 50/75/100 response by visit over time through Week 156 among the patients with total fingernail mNAPSI score >0 at baseline. - Proportion of patients who achieve PGA-F score of 0 [cleared]/1 [minimal] AND ≥2-grade reduction from baseline by visit over time through Week 156 among the patients with PGA-F score ≥2 at baseline.   **Endpoints Related to Joint Structural Damage**   - Change from baseline in modified vdH-S score by visit over time through Week 156 and change from Week 24 to Weeks 48, 96, 156; from Week 48 to Weeks 96 and 156; and from Week 96 to Week 156. - Change from baseline in modified vdH-S erosion score and JSN score by visit over time through Week 156 and from Week 24 to Weeks 48, 96, 156; from Week 48 to Weeks 96 and 156; and from Week 96 to Week 156. - Change from baseline in modified vdH-S score by region and type of damage (i.e., hand erosion, hand JSN, foot erosion, foot JSN subscores) by visit over time through Week 156. - Proportion of patients with a change of ≤0 from baseline, and proportion of patients with a change of ≤0.5 from baseline in modified vdH-S score or modified vdH-S erosion score and JSN score by visit over time through Week 156. - Proportion of patients with radiographic progression (based on the SDC) or radiographic joint erosion progression and radiographic JSN progression (based on SDC) from baseline by visit over time through Week 156. - Proportion of patients with pencil in cup or gross osteolysis deformities by visit over time through Week 156.   **Endpoints Related to Health-Related Quality of Life**   - Change from baseline in FACIT-F score by visit over time through Week 156. - Proportion of patients who achieve ≥4-point improvement from baseline in FACIT-F score improvement by visit over time through Week 156. - Change from baseline in PsAID-12 by visit over time through Week 156.   **Endpoint Related to Pharmacodynamic effect**   - Change from baseline in serum interleukin (IL-17) cytokines by visit over time through Week 48. | |
| *ACR20/50/70 = American College of Rheumatology 20%/50%/70% improvement; AEs = adverse events; BSA = body surface area; CRP = C-reactive protein; CTCAE = Common Terminology Criteria for Adverse Events; DAPSA = Disease Activity Index for Psoriatic Arthritis; DAS = Disease Activity Score;; DLQI = Dermatology Life Quality Index; FACIT-F = Functional Assessment of Chronic Illness Therapy-Fatigue; HAQ-DI = Disability Index of the Health Assessment Questionnaire; IGA = Investigator’s Global Assessment; IL = interleukin; JSN = joint space narrowing; LEI = Leeds Enthesitis Index; mCPDAI = modified Composite Psoriatic Disease Activity Index; MDA = minimal disease activity; mNAPSI = modified Nail Psoriasis Severity Index; mPsARC = modified Psoriatic Arthritis Response Criteria; PASI75/90/100 = ≥75/90/100 improvement in Psoriatic Area and Severity Index; PGA-F = Physician’s Global Assessment of Fingernails Psoriasis; PK = pharmacokinetics; PsA = Psoriatic Arthritis; PsAID = Psoriatic Arthritis Impact of Disease; SAEs = serious adverse events; SDC = smallest detectable change; vdH-S = van der Heijde-Sharp; VLDA= very low disease activity; WPAI = Work Productivity and Activity Impairment.* | |
